# Efficacy of Orthokeratology in Controlling Axial Elongation in Myopic Children and Adolescents: A Systematic Review and Meta-Analysis of Comparative Interventions

**DOI:** 10.3390/jcm15155776

**Published:** 2026-07-23

**Authors:** António Queirós, Inês Mota-Silva, Inês Pinheiro

**Affiliations:** 1 Clinical and Experimental Optometry Research Lab (CEORLab), School of Science, University of Minho, 4710-057 Braga, Portugal; ismota20@gmail.com (I.M.-S.); id12113@alunos.uminho.pt (I.P.); 2 Physics Center of Minho and Porto Universities, CF-UM-UP, 4710-057 Braga, Portugal

**Keywords:** orthokeratology, myopia control, axial length, children, meta-analysis, atropine, multifocal contact lenses

## Abstract

**Background**: Myopia represents a growing global public health concern, with prevalence projected to reach 4.9 billion people by 2050. Orthokeratology has emerged as a non-invasive optical intervention for myopia control in children. This systematic review and meta-analysis evaluated the efficacy of orthokeratology in reducing axial length (AL) growth compared to spectacles, atropine combinations, multifocal contact lenses, gradient soft contact lenses, modified orthokeratology with enhanced peripheral defocus (enhanced OK), and atropine therapy with single-vision spectacle correction. **Methods**: A systematic literature search was conducted in PubMed and Google Scholar for studies published in the past 20 years (2004–2024) using keywords “orthokeratology” OR “corneal reshaping” AND “myopia control.” Inclusion criteria comprised longitudinal clinical studies (randomized or non-randomized) with AL measurements, at least two groups including orthokeratology, and publication in English or Portuguese. Meta-analysis was performed using Review Manager 5.0.25 with random-effects models. Heterogeneity was assessed using I^2^ statistics, and subgroup analyses were conducted by follow-up duration. **Results**: Thirty-one studies met inclusion criteria. Orthokeratology significantly reduced AL growth compared to spectacles (25 studies, 4267 patients; mean difference [MD] = −0.16 mm, 95% CI −0.19 to −0.14, *p* < 0.00001, I^2^ = 97%). Combined orthokeratology with atropine showed superior efficacy to orthokeratology alone (8 studies, 888 patients; MD = 0.09 mm, 95% CI 0.06 to 0.12, *p* < 0.00001, I^2^ = 98%). Orthokeratology demonstrated equivalent efficacy to multifocal contact lenses (3 studies, 208 patients; MD = 0.00 mm, 95% CI −0.04 to 0.05, *p* = 0.85, I^2^ = 83%). Atropine therapy with single-vision spectacle correction and enhanced OK showed superior myopia control compared to conventional orthokeratology. **Conclusions**: Orthokeratology effectively slows axial elongation in myopic children compared to spectacles, with efficacy comparable to multifocal contact lenses. Combination therapies, particularly orthokeratology with atropine, demonstrate additive benefits. High heterogeneity across studies highlights the need for standardized protocols and long-term follow-up studies to optimize myopia control strategies.

## 1. Introduction

Myopia, or nearsightedness, has emerged as one of the most significant public health challenges of the 21st century, with prevalence rates increasing dramatically across all continents, particularly in East Asian populations [1,2]. Current epidemiological projections estimate that approximately 2.6 billion people worldwide were affected by myopia in 2020, with this number expected to rise to 4.9 billion by 2050, representing nearly half of the global population [3]. This escalating prevalence is particularly concerning in pediatric populations, where early onset and rapid progression increase the risk of developing high myopia and associated sight-threatening complications in adulthood [4].

High myopia, typically defined as a spherical equivalent refractive error of ≤−6.00 diopters (D) or axial length greater than 26 mm, is associated with increased risk of pathological ocular conditions including myopic maculopathy, retinal detachment, glaucoma, and cataracts [5,6]. The relationship between axial elongation and these complications is well-established, with each millimeter increase in axial length associated with a 67% increased risk of myopic maculopathy [7]. Consequently, interventions that slow axial elongation during childhood have the potential to reduce the lifetime risk of vision-threatening complications and associated healthcare costs [8].

The mechanisms underlying myopia development and progression involve complex interactions between genetic predisposition, environmental factors, and optical signaling pathways that regulate eye growth [9]. Current evidence suggests that peripheral hyperopic defocus plays a critical role in stimulating axial elongation, while peripheral myopic defocus may slow eye growth [8,10]. This understanding has led to the development of optical interventions designed to manipulate peripheral retinal defocus, including orthokeratology [11], multifocal contact lenses [12], and defocus-incorporated spectacle lenses [13]. 

Orthokeratology involves the overnight wear of specially designed rigid gas-permeable contact lenses that temporarily reshape the corneal surface, providing clear daytime vision without the need for daytime optical correction while simultaneously inducing peripheral myopic defocus [14,15]. The proposed mechanism of myopia control through orthokeratology involves central corneal flattening combined with mid-peripheral corneal steepening, which creates a myopic defocus signal in the peripheral retina that is hypothesized to slow axial elongation [16]. Clinical studies have demonstrated that orthokeratology can reduce the rate of axial elongation by approximately 30–60% compared to single-vision spectacles [17,18,19].

Pharmacological interventions, particularly topical atropine, represent an alternative approach to myopia control. Atropine, a non-selective muscarinic antagonist, has demonstrated dose-dependent efficacy in slowing myopia progression, with higher concentrations (0.5–1.0%) showing greater efficacy but also increased side effects including photophobia and accommodation loss [20]. Low-dose atropine (0.01%) has emerged as a promising option with fewer side effects and sustained efficacy, though slightly lower than higher concentrations [20,21,22]. Recent studies have explored combination therapies, particularly orthokeratology with low-dose atropine, which may provide additive benefits in myopia control [23].

Multifocal contact lenses and spectacles represent additional optical strategies for myopia control. Multifocal soft contact lenses with peripheral add power have demonstrated efficacy in slowing axial elongation, with effect sizes comparable to orthokeratology in some studies [24]. Defocus Incorporated spectacle lenses have also shown promise in clinical trials, offering a non-contact lens option for myopia control [13,25,26].

Despite the growing body of evidence supporting various myopia control interventions, comparative effectiveness data remain limited, and optimal treatment selection for individual patients remains unclear. Previous meta-analyses have primarily focused on comparing single interventions to control groups, with limited direct comparisons between active treatments. Furthermore, the relative efficacy of combination therapies compared to monotherapy requires systematic evaluation. The aim of this systematic review and meta-analysis was to comprehensively evaluate the efficacy of orthokeratology in controlling axial elongation in myopic children and adolescents compared to alternative interventions, including spectacles, atropine combinations, multifocal contact lenses, gradient soft contact lenses, modified orthokeratology with enhanced peripheral defocus (enhanced OK), and spectacles combined with atropine. By synthesizing data from studies published over the past 20 years, this analysis provides evidence-based guidance for clinicians and researchers regarding the comparative effectiveness of available myopia control strategies.

## 2. Materials and Methods

### 2.1. Search Strategy

**Figure 1:**
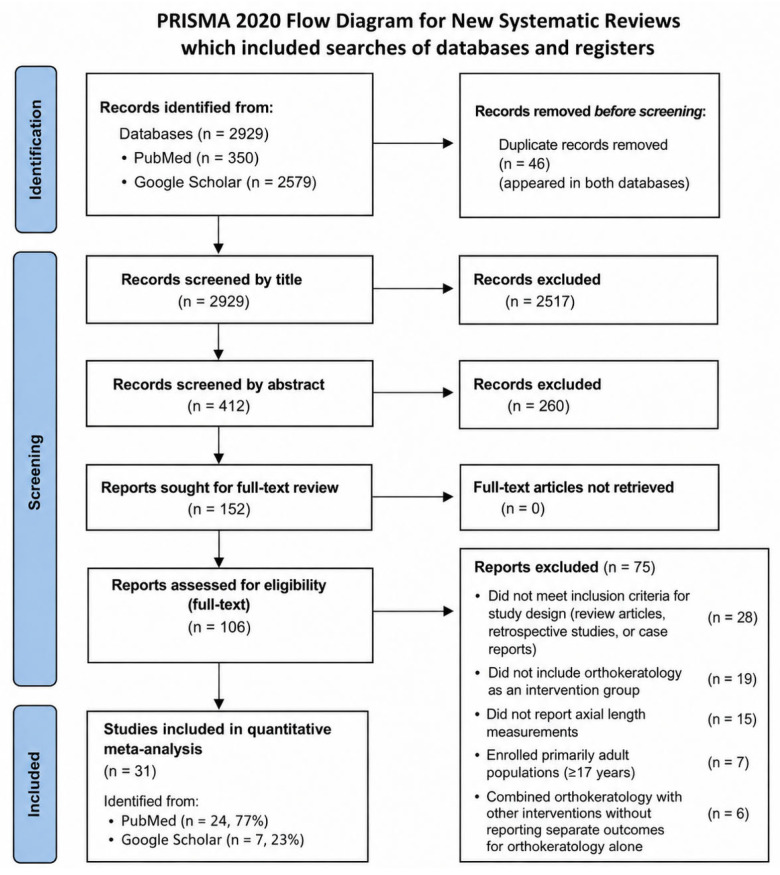
PRISMA flow diagram of the literature search and study selection process. A total of 2929 records were identified through database searching (PubMed, *n* = 350; Google Scholar, *n* = 2579). Following title screening, abstract screening, duplicate removal, and full-text assessment, 31 studies met the inclusion criteria and were included in the meta-analysis.

A comprehensive systematic literature search was conducted to identify studies evaluating the efficacy of orthokeratology for myopia control in children and adolescents. Two major electronic databases were searched: PubMed (MEDLINE) and Google Scholar. The search was limited to articles published within the last 20 years (2004–2024) to capture contemporary evidence using modern orthokeratology lens designs and standardized outcome measures. The search strategy employed the following keywords and Boolean operators: “orthokeratology” OR “corneal reshaping” OR “corneal refractive therapy” OR “reverse geometry lenses” AND “myopia control” OR “myopia progression” OR “axial elongation”. These terms were selected to capture studies investigating orthokeratology as an intervention for slowing myopia progression, while excluding studies focused solely on refractive correction without myopia control outcomes. The initial search was conducted in January 2024 and yielded 2929 articles: 350 from PubMed and 2579 from Google Scholar. All identified articles underwent systematic screening through multiple stages, as described in the PRISMA flow diagram (Figure 1 and Table S1).

### 2.2. Inclusion and Exclusion Criteria

Inclusion Criteria: Studies were included if they met all of the following criteria. Study design: Longitudinal clinical studies, including both randomized controlled trials and non-randomized comparative studies. Prospective cohort studies with appropriate control groups were eligible. Outcome measures: Studies must have reported axial length measurements or vitreous chamber depth as primary or secondary outcomes. Studies reporting only refractive error changes without biometric measurements were excluded. Intervention groups: Studies must have included at least two groups, with one group receiving orthokeratology treatment. The comparison group could include spectacles, atropine (alone or combined with orthokeratology), multifocal contact lenses, or other myopia control interventions. Population: Studies must have enrolled children or adolescents (age <17 years at baseline) with myopia. Studies including young adults (≥17 years) as the primary population were excluded. Language: Articles published in English were eligible for inclusion. Publication type: Full-text peer-reviewed journal articles were required. Conference abstracts, dissertations, and unpublished data were excluded.

Exclusion Criteria: Studies were excluded if they met any of the following criteria: Review articles—Systematic reviews, meta-analyses, narrative reviews, and editorial commentaries were excluded. However, reference lists of relevant reviews were hand-searched to identify additional primary studies. Age criteria: Studies enrolling primarily young adults (≥17 years at baseline) were excluded, as myopia progression patterns and treatment responses differ in adult populations. Retrospective design: Retrospective chart reviews and case series were excluded due to higher risk of bias and inability to establish temporal relationships between intervention and outcomes. Combined interventions without separate analysis: Studies that combined orthokeratology with other myopia control interventions (e.g., orthokeratology plus atropine) without reporting outcomes for orthokeratology alone were excluded from comparisons of orthokeratology monotherapy but were included in separate analyses of combination therapies. Insufficient data: Studies that did not report sufficient data for meta-analysis (mean axial length change, standard deviation, and sample size for each group) were excluded if authors could not be contacted or did not provide additional data upon request.

Myopia-control spectacle lenses, including Defocus Incorporated Multiple Segments (DIMS) spectacles and Highly Aspherical Lenslet Target (HALT) lenses, were included as eligible comparison interventions. However, no eligible studies directly comparing orthokeratology to myopia-control spectacle lenses with axial length as the primary outcome were identified within the search period.

### 2.3. Data Extraction

Two independent reviewers (AQ and IM) screened titles and abstracts of all identified articles against the inclusion and exclusion criteria. Articles deemed potentially eligible by either reviewer proceeded to full-text review. Full-text articles were independently assessed by both reviewers, with disagreements resolved through discussion or consultation with a third reviewer when necessary. For each included study, the following data were systematically extracted using a standardized data extraction form—Study characteristics: First author, publication year, country, study design (randomized controlled trial vs. non-randomized comparative study), follow-up duration, and sample size. Participant characteristics: Number of participants in each group, mean age and standard deviation, sex distribution, baseline refractive error (spherical equivalent), and baseline axial length. Intervention details: For orthokeratology groups, lens design, material, oxygen permeability (Dk value), target correction, and wearing schedule. For comparison groups, details of spectacle correction, atropine concentration and dosing regimen, or contact lens specifications. Outcome data: Mean change in axial length from baseline at each reported time point (1, 3, 6, 12, 18, and 24 months), with corresponding standard deviations and sample sizes. When studies reported multiple time points, data from all available time points were extracted for subgroup analyses. Additional outcomes: Secondary outcomes including refractive error change, corneal curvature changes, and adverse events were recorded when reported. Data extraction was performed independently by two reviewers, with discrepancies resolved through discussion and reference to the original articles. When data were presented only in graphical form, digital plot digitizer software (WebPlotDigitizer software, v4) was used to extract numerical values, with verification by both reviewers.

### 2.4. Statistical Analysis

Meta-analysis was conducted using Review Manager software (RevMan Version 5.0.25, The Cochrane Collaboration, Oxford, UK). The primary outcome measure was the mean difference in axial length change between orthokeratology and comparison groups, expressed in millimeters with 95% confidence intervals. Effect Size Calculation: For each study, the mean difference in axial length change between groups was calculated as MD = (Mean AL change in OK group) − (Mean AL change in comparison group). A negative mean difference indicates that orthokeratology resulted in less axial elongation (i.e., better myopia control) compared to the comparison group, while a positive mean difference indicates greater axial elongation with orthokeratology. Standard errors were calculated from reported standard deviations and sample sizes. When studies reported standard errors or confidence intervals instead of standard deviations, appropriate conversions were performed. Meta-Analytic Model: Given the expected heterogeneity in study populations, intervention protocols, and follow-up durations, a random-effects model was used for all meta-analyses. The random-effects model assumes that true effect sizes vary across studies and provides more conservative estimates of precision compared to fixed-effect models. The DerSimonian and Laird method was used to estimate between-study variance (τ^2^). Pooled effect estimates were calculated as weighted means of individual study effects, with weights inversely proportional to the variance of each study’s effect estimate. Heterogeneity Assessment: Statistical heterogeneity was assessed using the I^2^ statistic, which represents the percentage of total variation across studies that is due to heterogeneity rather than chance. I^2^ values were interpreted according to the following thresholds: 0–40%: might not be important; 30–60%: may represent moderate heterogeneity; 50–90%: may represent substantial heterogeneity; 75–100%: considerable heterogeneity. The Chi-squared test for heterogeneity was also performed, with *p* < 0.10 indicating statistically significant heterogeneity. Subgroup Analyses: To explore sources of heterogeneity and assess whether treatment effects varied by follow-up duration, subgroup analyses were conducted stratifying studies by time point: <3 months; 3 months; 6 months; 12 months; 18 months; and 24 months. For studies reporting multiple time points, each time point was included as a separate comparison in the subgroup analysis. Tests for subgroup differences were performed using the Chi-squared test. Comparison Groups: Separate meta-analyses were conducted for each of the following comparisons: Orthokeratology versus single-vision spectacles; Orthokeratology versus orthokeratology combined with atropine; Orthokeratology versus multifocal contact lenses; Orthokeratology versus gradient soft contact lenses; Orthokeratology versus enhanced OK; and Orthokeratology versus atropine therapy with single-vision spectacle correction. An overall comparison combining all intervention types was also performed to assess the general position of orthokeratology relative to all other myopia control strategies. Publication Bias Assessment: Publication bias was assessed visually using funnel plots, which display effect estimates from individual studies against their standard errors. In the absence of publication bias, studies should be distributed symmetrically around the pooled effect estimate, forming an inverted funnel shape. Asymmetry in funnel plots may indicate publication bias, though it can also result from heterogeneity or chance. For comparisons including 10 or more studies, formal statistical tests for funnel plot asymmetry (Egger’s test) were considered, though interpretation was cautious given the limitations of these tests in the presence of heterogeneity. Sensitivity Analyses: Sensitivity analyses were planned to assess the robustness of findings, including restriction to randomized controlled trials only; exclusion of studies with high risk of bias; and exclusion of outlier studies contributing to heterogeneity. However, given the limited number of studies in some comparison groups, formal sensitivity analyses were only performed for the orthokeratology versus spectacles comparison.

## 3. Results

### 3.1. Study Selection

The systematic literature search identified 2929 articles: 350 from PubMed and 2579 from Google Scholar (Figure 1). After initial screening based on titles, 412 articles were retained for abstract review. Following abstract screening, 152 articles proceeded to full-text review. After removing 46 duplicate articles that appeared in both databases, 106 unique articles underwent detailed full-text assessment. During the final screening stage, 75 articles were excluded for the following reasons: 28 did not meet inclusion criteria for study design (review articles, retrospective studies, or case reports), 19 did not include orthokeratology as an intervention group, 15 did not report axial length measurements, 7 enrolled primarily adult populations (≥17 years), and 6 combined orthokeratology with other interventions without reporting separate outcomes for orthokeratology alone. Ultimately, 31 studies met all inclusion criteria and were included in the quantitative meta-analysis. Of these, 24 studies (77%) were identified through PubMed and 7 studies (23%) through Google Scholar. The included studies were published between 2005 and 2024, with the majority (*n* = 23, 74%) published after 2015, reflecting the recent growth in myopia control research.

### 3.2. Orthokeratology Versus Spectacles

**Figure 2:**
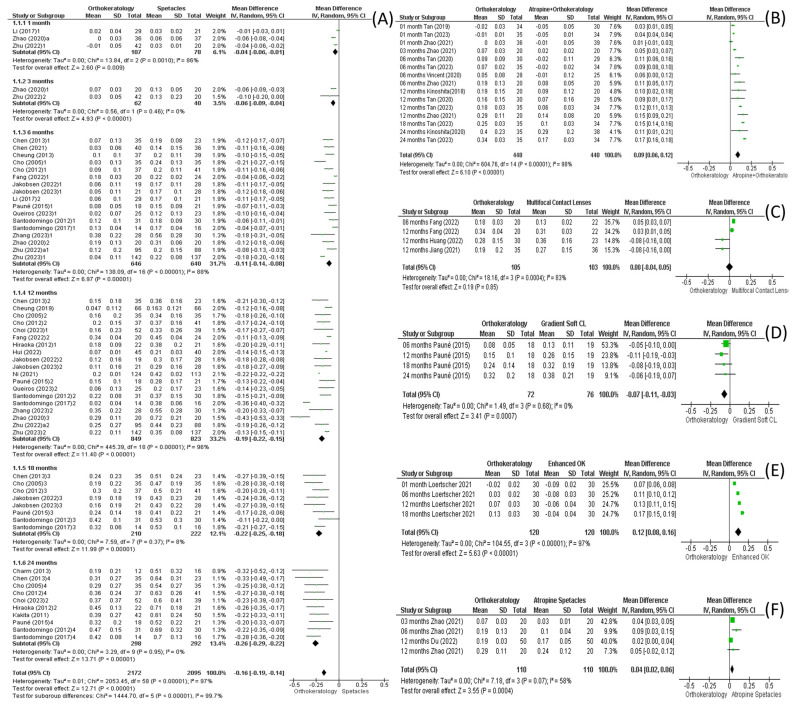
Forest plots of meta-analyses evaluating the effectiveness of orthokeratology and related interventions for myopia control based on axial length changes. (**A**) Orthokeratology versus single-vision spectacles; (**B**) orthokeratology versus orthokeratology combined with 0.01% atropine; (**C**) orthokeratology versus multifocal soft contact lenses; (**D**) orthokeratology versus gradient soft contact lenses; (**E**) conventional versus modified orthokeratology with enhanced peripheral defocus (enhanced OK); and (**F**) orthokeratology versus atropine therapy with single-vision spectacle correction. Mean differences (MDs) and 95% confidence intervals (CIs) are presented for each comparison. Green squares represent the effect estimates of the individual studies (with square size proportional to study weight), and black diamonds represent the pooled effect estimates with their 95% confidence intervals. All studies identified by the “Author (Year)” labels in the forest plots are fully referenced in Table 1, Table 2, Table 3, Table 4, Table 5 and Table 6 [17,18,23,27,28,29,30,31,32,33,34,35,36,37,38,39,40,41,42,43,44,45,46,47,48,49,50,51,52,53,54,55,56,57,58].

Twenty-five studies compared orthokeratology to single-vision spectacles for myopia control, representing the largest body of comparative evidence (Table 1). These studies included a total of 4267 participants: 2172 in orthokeratology groups and 2095 in spectacle control groups. The mean age of participants across studies was 10.23 ± 1.21 years, with baseline myopia ranging from −0.75 D to −5.00 D across studies. Follow-up durations varied across studies, with data available at multiple time points: 3 studies reported outcomes at 1 month, 2 studies at 3 months, 17 studies at 6 months, 19 studies at 12 months, 8 studies at 18 months, and 10 studies at 24 months. Several studies contributed data at multiple time points, allowing for subgroup analyses by follow-up duration. The pooled meta-analysis demonstrated that orthokeratology significantly reduced axial elongation compared to spectacles (Figure 2). The overall mean difference in axial length change was −0.16 mm (95% CI: −0.19 to −0.14 mm, *Z* = 12.71, *p* < 0.00001), indicating that children wearing orthokeratology lenses experienced 0.16 mm less axial elongation compared to those wearing spectacles over the study periods. This difference was highly statistically significant and clinically meaningful. However, substantial heterogeneity was observed across studies (I^2^ = 97%, *p* < 0.00001), indicating considerable variation in treatment effects. This heterogeneity likely reflects differences in study populations (age, baseline myopia, ethnicity), orthokeratology lens designs, follow-up durations, and study quality.

**Figure 3:**
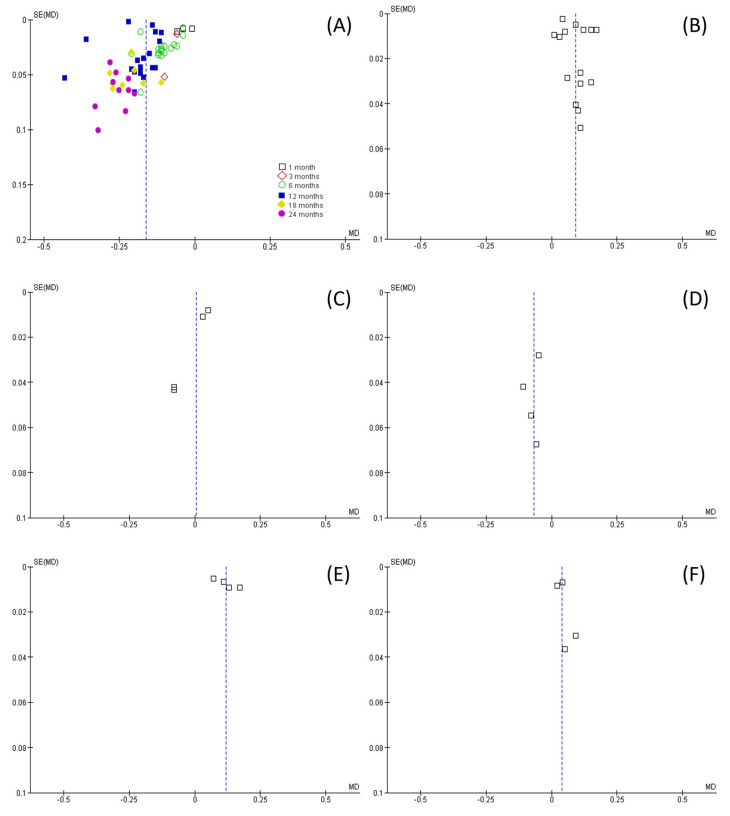
Funnel plots assessing potential publication bias for studies included in the meta-analyses of myopia control interventions. (**A**) Orthokeratology versus single-vision spectacles; (**B**) orthokeratology versus orthokeratology combined with 0.01% atropine; (**C**) orthokeratology versus multifocal soft contact lenses; (**D**) orthokeratology versus gradient soft contact lenses; (**E**) conventional versus modified orthokeratology with enhanced peripheral defocus (enhanced OK); and (**F**) orthokeratology versus atropine therapy with single-vision spectacle correction. The blue dashed vertical line represents the pooled mean difference (overall effect estimate) for each meta-analysis.

Subgroup analysis by follow-up duration revealed important temporal patterns in treatment efficacy (Figure 2A). At less than 3 months of follow-up, the mean difference was −0.04 mm (95% CI: −0.06 to −0.01 mm, *Z* = 2.60, *p* = 0.009, I^2^ = 86%), indicating a small but statistically significant early effect. At 24 months, the mean difference increased to −0.26 mm (95% CI: −0.29 to −0.22 mm, *Z* = 13.71, *p* < 0.001, I^2^ = 0%), demonstrating greater cumulative benefit with longer treatment duration and notably reduced heterogeneity at this time point. The test for subgroup differences was statistically significant (*p* < 0.001), confirming that treatment effects varied significantly by follow-up duration. The increasing magnitude of effect with longer follow-up reflects the cumulative nature of axial length differences, with orthokeratology consistently slowing but not completely halting axial elongation. Funnel plot analysis for this comparison showed relatively symmetric distribution of studies around the pooled effect estimate, suggesting no major publication bias (Figure 3A). However, some scatter was observed, particularly among smaller studies, which may reflect the substantial heterogeneity in treatment effects or small-study effects.

### 3.3. Orthokeratology Versus Orthokeratology Combined with Atropine

Eight studies compared orthokeratology alone to orthokeratology combined with atropine (Table 2). These studies included 888 participants: 448 receiving orthokeratology alone and 440 receiving combination therapy. The mean age was 10.26 ± 0.26 years, similar to the orthokeratology versus spectacles comparison. All studies used low-dose atropine (0.01%) in combination with orthokeratology, reflecting current clinical practice favoring low-dose formulations.

Follow-up periods ranged from 1 to 24 months, with studies reporting outcomes at 1, 3, 6, 12, 18, and 24 months. The meta-analysis demonstrated that combination therapy with orthokeratology plus atropine was significantly more effective than orthokeratology alone in slowing axial elongation (Figure 2B). The pooled mean difference was 0.09 mm (95% CI: 0.06 to 0.12 mm, *Z* = 6.10, *p* < 0.00001), indicating that orthokeratology alone resulted in 0.09 mm more axial elongation compared to combination therapy. The positive mean difference reflects that orthokeratology alone was less effective than the combination, with combination therapy providing superior myopia control. Substantial heterogeneity was observed (I^2^ = 98%, *p* < 0.00001), likely reflecting differences in follow-up duration, baseline myopia severity, and patient characteristics across studies. Despite this heterogeneity, the direction of effect was consistent across all studies, with every included study showing numerically greater myopia control with combination therapy. The magnitude of additional benefit from adding atropine to orthokeratology (approximately 0.09 mm over study periods ranging from 6 to 24 months) represents a clinically meaningful enhancement. Assuming an average follow-up of 18 months across studies, this translates to an additional reduction in axial elongation of approximately 0.06 mm per year with combination therapy.

Funnel plot analysis showed reasonable symmetry, though the small number of studies limits interpretation of publication bias (Figure 3B). The consistency of effect direction across all studies, despite heterogeneity in effect magnitude, provides confidence in the conclusion that combination therapy offers additive benefit.

### 3.4. Orthokeratology Versus Multifocal Contact Lenses

Three studies directly compared orthokeratology to multifocal soft contact lenses for myopia control (Table 3), including a total of 208 participants with a mean age of 12.07 ± 1.94 years. The multifocal contact lenses evaluated in these studies included various designs with peripheral add powers ranging from +1.50 D to +2.50 D. The meta-analysis revealed no significant difference in axial length control between orthokeratology and multifocal contact lenses (Figure 2C). The pooled mean difference was 0.00 mm (95% CI: −0.04 to 0.05 mm, *Z* = 0.19, *p* = 0.85), indicating equivalent efficacy between these two optical interventions. Substantial heterogeneity was present (I^2^ = 83%, *p* = 0.003), likely reflecting differences in multifocal lens designs, add powers, and patient populations across the three studies. Despite this heterogeneity, the confidence interval for the pooled effect was narrow and centered on zero, providing reasonable confidence in the conclusion of equivalent efficacy. This finding has important clinical implications, as it suggests that both orthokeratology and multifocal contact lenses represent effective optical strategies for myopia control, with similar efficacy in slowing axial elongation. The choice between these interventions can therefore be guided by patient preferences, lifestyle factors, and individual suitability rather than expected efficacy differences. The funnel plot for this comparison (Figure 3C) showed limited scatter given the small number of studies, with no obvious asymmetry. However, the small sample size limits conclusions regarding publication bias. It should be noted that gradient soft contact lenses and multifocal soft contact lenses share a similar optical design principle: both create peripheral myopic defocus intended to slow axial elongation. Gradient designs achieve this through a continuous increase in positive power from center to periphery, while multifocal designs use discrete optical zones with defined add powers. Despite this design similarity, the two comparisons are presented separately given their distinct study populations, follow-up durations, and the limited evidence base (one study for gradient SCL versus three studies for multifocal SCL). Readers are encouraged to consider these comparisons together when interpreting the overall efficacy of soft contact lens-based myopia control strategies.

### 3.5. Orthokeratology Versus Gradient Soft Contact Lenses

As noted in Section 3.4, gradient soft contact lenses share a similar optical design principle with multifocal soft contact lenses: both create peripheral myopic defocus through manipulation of the lens power profile. These two comparisons should be considered together when interpreting the overall efficacy of soft contact lens-based strategies.

One study (Paune et al. [36], 2015; *n* = 18 participants) compared orthokeratology to gradient soft contact lenses (also known as peripheral gradient contact lenses), reporting outcomes at multiple time points: 6, 12, 18, and 24 months (Table 4). The study included participants with a mean age of 12.27 ± 1.76 years. Gradient soft contact lenses feature a gradual increase in plus power from the center to the periphery, designed to create peripheral myopic defocus similar to multifocal designs but with a continuous gradient rather than discrete zones. The meta-analysis of data from multiple time points within this single study demonstrated that orthokeratology was significantly more effective than gradient soft contact lenses in controlling axial elongation (Figure 2D). The pooled mean difference was −0.07 mm (95% CI: −0.11 to −0.03 mm, *Z* = 3.41, *p* = 0.0007), indicating that orthokeratology resulted in 0.07 mm less axial elongation compared to gradient soft contact lenses. Notably, heterogeneity was absent across the different time points (I^2^ = 0%, *p* = 0.95), indicating consistent treatment effects throughout the 24-month follow-up period. Since all data originated from a single study, this I^2^ value reflects within-study temporal consistency rather than between-study heterogeneity and should not be interpreted in the conventional meta-analytic sense. While this comparison is limited by being based on a single study, the consistent treatment effect across all four follow-up time points supports the internal consistency of this finding. It must be emphasized, however, that consistency across time points within a single study cannot substitute for independent replication in different populations and study settings. Confirmation in additional trials is required before definitive conclusions can be drawn. As this comparison is based on a single study with repeated measurements at different time points, funnel plot analysis (Figure 3D) cannot provide information about publication bias. Figure 3D is retained for visual completeness but does not constitute evidence regarding publication bias.

### 3.6. Orthokeratology Versus Modified Orthokeratology with Enhanced Peripheral Defocus 

Terminological note: The term ‘modified orthokeratology with enhanced peripheral defocus (enhanced OK)’ used in the original study (Loertscher et al., 2021) [57] refers to orthokeratology lenses with a modified design intended to produce greater peripheral myopic defocus compared to conventional designs. As all orthokeratology lenses inherently create a multifocal corneal refractive profile, this term is potentially misleading. Throughout this manuscript, we use the more descriptive term ‘modified orthokeratology with enhanced peripheral defocus’ (enhanced OK) to refer to this lens category.

One study (Loertscher et al., 2021 [57]; *n* = 30 participants) compared conventional orthokeratology to enhanced OK, reporting outcomes at 1, 6, 12, and 18 months (Table 5). The study included participants with a mean age of 12.20 ± 1.3 years. Enhanced OK incorporates additional design features intended to enhance peripheral myopic defocus beyond that achieved with conventional orthokeratology designs. The meta-analysis demonstrated that enhanced OK was significantly more effective than conventional orthokeratology in controlling axial elongation (Figure 2E). The pooled mean difference was 0.12 mm (95% CI: 0.08 to 0.16 mm, *Z* = 5.63, *p* < 0.00001), indicating that conventional orthokeratology resulted in 0.12 mm more axial elongation compared to enhanced OK. Substantial variability was observed across time points (I^2^ = 97%, *p* < 0.00001), reflecting increasing treatment effect magnitude over follow-up. Since all data originated from a single study, this I^2^ value reflects within-study temporal variability rather than between-study heterogeneity and should not be interpreted as conventional meta-analytic heterogeneity. The magnitude of difference between conventional and enhanced OK (0.12 mm) is clinically meaningful and suggests that design modifications to enhance peripheral defocus may provide additional myopia control benefit. However, as with the gradient soft contact lens comparison, this finding is based on a single study and requires replication in additional trials. The high heterogeneity across time points also suggests that the treatment effect may vary over the course of treatment, warranting further investigation. Similarly, as this comparison is also based on a single study with repeated measurements, funnel plot analysis (Figure 3E) cannot provide information about publication bias. Figure 3E is retained for completeness but does not constitute evidence regarding publication bias.

### 3.7. Orthokeratology Versus Atropine Therapy with Single-Vision Spectacle Correction

Terminological note: In this comparison, the spectacle correction worn alongside atropine serves only as refractive correction; the myopia-control effect is attributable solely to atropine. This group is therefore best described as atropine monotherapy with standard refractive correction, rather than a ‘combined’ therapy.

Two studies compared orthokeratology to atropine therapy with single-vision spectacle correction (Table 6), including 220 participants: 110 receiving orthokeratology and 110 receiving spectacles plus atropine. The mean age was 10.23 ± 1.00 years. Both studies used low-dose atropine (0.01%) combined with single-vision spectacles. Follow-up periods included 3, 6, and 12 months. The meta-analysis demonstrated that atropine therapy with single-vision spectacle correction was significantly more effective than orthokeratology alone in controlling axial elongation (Figure 2F). The pooled mean difference was 0.04 mm (95% CI: 0.02 to 0.06 mm, *Z* = 3.55, *p* = 0.0004), indicating that orthokeratology alone resulted in 0.04 mm more axial elongation compared to atropine therapy with single-vision spectacle correction. Moderate heterogeneity was observed (I^2^ = 58%, *p* = 0.12), which may reflect differences in follow-up duration and patient characteristics between the two studies. The magnitude of difference (0.04 mm) is smaller than that observed when comparing orthokeratology alone to orthokeratology plus atropine (0.09 mm), suggesting that the myopia control efficacy of orthokeratology alone is intermediate between spectacles alone and atropine therapy with single-vision spectacle correction. This comparison provides important context for understanding the relative efficacy of different myopia control strategies. The finding that atropine therapy with single-vision spectacle correction outperforms orthokeratology alone, while orthokeratology plus atropine outperforms orthokeratology alone, suggests that atropine provides consistent additive benefit regardless of the optical correction method. The funnel plot for this comparison (Figure 3F) showed limited scatter given the small number of studies, with no obvious asymmetry.

**Table 1:** Summary of studies included in the orthokeratology versus spectacle correction meta-analysis.

Author, Year	Study Type, Time	Etnia, Simple Size	Brand	Material	Dk *	Overall Diameter (mm)	Optic Zone (mm)	Geometry	Outcome Assessment Method	Age Mean ± SD (Years)
Charm, 2013 [27]	Randomized controlled clinical trial, 24 months	Chinese, 12	Procornea Ltd., Netherlands	Hexafocon A	100	10.5	6.0	four-zone reverse geometry	Open-field autorefractor Shin-Nippon K 5001 noncycloplegic and cycloplegic IOL Master	10 (8 to11)
Chen, 2013 [28]	Nonrandomized clinical study, 24 months	Chinese, 35	NKL Contactlenzen B.V., Emmen	hyper-permeable copolymer of siloxanyl styrene and fluoromethacrylate	163	10.2/10.6/11	6.0	Parallel reverse	Cycloplegic IOL Master	9.4 ± 1.4
Cho, 2005 [29]	x	Chinese, 35	x	x	x	x	x	x	x A-scan ultrasonography	9.57 ± 1.46
Cho, 2012 [17]	Randomized controlled clinical trial, 24 months	Chinese, 237	NKL Contactlenzen B.V., Emmen	hyper-permeable copolymer of siloxanyl styrene and fluoromethacrylate	163	10.2/10.6/11	x	x	x IOL Master	9.23 ± 1.06
Choi, 2023 [30]	Randomized controlled clinical trial, 24 months	Chinese, 52	Universal View Co., Ltd.	Silicone and Fluorine methacrylate compounds	156	10.2–11.0	x	four-zone reverse geometry	Open-field autorefractor Shin-Nippon K 5001 cycloplegic autorefraction IOL Master 500	9.8 ± 1.3
Fang, 2022 [31]	Parallel, longitudinal, single-blind, randomized clinical trial, 12 months	Chinese, 20	Paragon CRT, Paragon, Gilbert, AZ)	x	x	9.5–12	5.0/6.0	x	Humphrey Autorefractor Keratometer HARK599 cycloplegic autorefraction IOL Master 700	12.5 ± 0.3
Jakobsen 2022 [32]	Randomized controlled clinical trial, 18 months	Scandinavian, 19	Pro-cornea, LZ Eerbeek, the Netherlands	hexafocon A	100	x	6.0	four-zone reverse geometry	Open-field autorefractor Shin-Nippon K 5001 cycloplegic autorefraction Lenstar LS 900	10.03 ± 1.6
Jakobsen, 2023 [33]	Randomized controlled clinical trial, 18 months	Danish, 21	Pro-cornea, LZ Eerbeek, the Netherlands	hexafocon A	100	x	6.0	four-zone reverse-geometry	Open-field autorefractor Shin-Nippon K 5001 cycloplegic autorefraction Lenstar LS 900	9.96 ± 1.54
Kakita, 2011 [34]	Randomized controlled clinical trial, 24 months	Japanese, 42	Bausch + Lomb	Oprifocona A	127	10.6	5.50–6.0	four-zone reverse-geometry	x IOL Master	12 ± 2.6
Li, 2017 [35]	Prospective, nonrandomized study, 6 months	Chinese, 29	Bausch + Lomb	Oprifocona A	127	10 to 10.8	5.50–6.0	four-zone reverse-geometry	Cycloplegic Lenstar LS 900	12.31 ± 1.71
Pauné, 2015 [36]	Prospective, longitudinal, nonrandomized study, 24 months	Spanish, 18	Precilens, Paris, France	Hexafocon B	141	10.00 to 11.60	x	x	Grand Seiko Autoref/Kerat WAM-5500 cycloplegic autorefraction A-scan ultrasonography	12.27 ± 1.76
Zhang, 2023 [37]	Randomized parallel-group, 12 months	Chinese, 28	Bausch + Lomb	Oprifocona A	127	10 to 10.8	5.50–6.0	four-zone reverse-geometry	Cycloplegic IOL Master	11
Zhao, 2021 [38]	Randomized controlled clinical trial, 12 months	Chinese, 20	x	x	x	x	x	x	Autorefractor TOPCON (KR-800) cycloplegic autorefraction Lenstar LS 900	11 ± 1.17
Zhao, 2021 [39]	Prospective, randomized controlled trial, 1 month	Chinese, 36	Bausch + Lomb	Oprifocona A	127	10.6–11.0	5.50–6.0	four-zone reverse-geometry	Autorefractor KR8800 cycloplegic autorefraction Lenstar LS 900	10.33 ± 1.65
Zhu, 2022 [40]	Prospective, nonrandomized controlled trial, 3 months	Chinese, 42	PARAGON CRT 100 Lens s (Paragon Vision Sciences, USA)	x	x	x	x	x	Noncycloplegic Lenstar LS 900	9.45 ± 1.37
Zhu, 2022 [41],	Longitudinal, prospective and nonrandomized study, 12 months	Chinese, 95	Bausch + Lomb	Oprifocona A	127	10.6–11.0	5.50–6.0	four-zone reverse geometry	Cycloplegic Lenstar LS 900	9.89 ± 1.99
Zhu, 2023 [42],	Prospective, nonrandomized controlled trial, 12 months	Chinese, 142	Bausch + Lomb	Oprifocona A	127	9.80–11.6	5.50–6.0	four-zone reverse geometry	Autorefractor KR8800 cycloplegic autorefraction IOL Master 700	9.18 ± 1.29
Santodomingo, 2017 [18]	Nonrandomized clinical study, 84 months	Spanish, 14	Menicon Co., Ltd., Nagoya, Japan	hyperpermeable copolymer of siloxanyl styrene and fluoromethacrylate	163	10.2/10.6/11	x	x	Autorefractor Topcon RM 8000B cycloplegic autorefraction IOL Master	10.4 ± 0.55
Santodomingo, 2012 [43]	Nonrandomized clinical study, 24 months	Spanish, 16	Menicon Co., Ltd., Nagoya, Japan	hyper-permeable copolymer of siloxanyl styrene and fluoromethacrylate	163	10.2/10.6/11	x	x	Autorefractor Topcon RM 8000B cycloplegic autorefraction IOL Master	9.6 ± 1.6
Chen, 2021 [44]	Prospective, nonrandomized controlled trial, 6 months	Chinese, 40	Customized lens (Lucid, Seoul, Korea)	x	x	x	x	x	Cycloplegic Lenstar LS 900	10 (9.00, 12.75)
Queirós, 2023 [45]	Nonrandomized clinical study, 12 months	French, 25	Precilens, Creteil, France	Hexafocon A	100	10.6	x	four-zone reverse geometry	AutoKerato-Refractometer TONOREF III cycloplegic autorefraction Lenstar LS 900	10.33 ± 1.25
Hiraoka, 2012 [46]	Prospective, nonrandomized controlled trial, 60 months	Japanese, 22	Euclid Systems Corp., Herndon, VA	hexafocon A	100	10.6	x	four-zone reverse geometry	x IOL Master	10.4 ± 1.43
Ni, 2021 [47]	Nonrandomized clinical study, 12 months	Chinese, 124	x	x	x	x	x	x	Cycloplegic IOL Master 500	11 ± 2.24
Hui, 2022 [48]	Prospective controlled trial, 12 months	Chinese, 45	ALPHA Corp., Nagoya, Japan	Silicone and Fluorine methacrylate compounds	104	10.5 mm	6.0	reverse geometry	x IOL Master	8–15
Cheung, 2013 [49]	Prospective, randomized study, 6 months	Chinese, 37	x	x	x	x	x	x	Cycloplegic IOL Master	9 (7–10)

* Dk (oxygen permeability, (10–11 cm^2^ × mL O_2_)/(s × mL × mmHg) or water content).

**Table 2:** Summary of studies included in the meta-analysis comparing orthokeratology and orthokeratology combined with low-dose atropine.

Author, Year	Study Type, Time	Etnia, Simple Size	Brand	Material	Dk *	Overall Diameter (mm)	Optic Zone (mm)	Geometry	Outcome Assessment Method	Age Mean ± SD (Years)
Tan, 2023 [23]	Single-masked, randomized study, 24 months	Chinese, 35	Precision Technology Services, Vancouver, B.C., Canada	HDS 100	100	10.2/10.6/ 11.0/11.2	6	four-zone reverse geometry	Cycloplegic IOL Master	9.1 ± 1.2
Tan, 2019 [50]	Randomized controlled clinical trial, 1 month	Chinese, 34	Precision Technology Services, Vancouver, B.C., Canada	HDS 100	100	10.2/10.6/ 11.0/11.2	6	four-zone reverse geometry	Cycloplegic IOL Master	9.09 ± 1.11
Vincent, 2020 [51]	Randomized controlled clinical trial, 6 months	Chinese, 28	Precision Technology Services, Vancouver, B.C., Canada	HDS 100	100	10.2/10.6/ 11.0/11.2	6	four-zone reverse geometry	Cycloplegic IOL Master 500	9.1 ± 1.1
Tan, 2020 [52]	Randomized controlled clinical trial, 12 months	Chinese,30	Precision Technology Services, Vancouver, B.C., Canada	HDS 100	100	10.2/10.6/ 11.0/11.2	6	four-zone reverse geometry	Cycloplegic IOL Master	9.0 ± 1.2
Kinoshita, 2020 [53]	Randomized controlled clinical trial, 24 months	Japanese, 35	Universal View Co., Ltd.	Silicone and Fluorine methacrylate compounds	156	10.2–11.0	x	four-zone reverse geometry	Cycloplegic IOL Master	10.37 ± 1.65
Kinoshita, 2018 [54]	Randomized controlled clinical trial, 12 months	Japanese, 20	Universal View Co., Ltd.	Silicone and Fluorine methacrylate compounds	156	10.2–11.0	x	four-zone reverse geometry	Cycloplegic IOL Master	10.4 ± 1.86
Zhao, 2021 [39]	Prospective, randomized controlled trial, 1 month	Chinese, 36	Bausch + Lomb	Oprifocona A	127	9.80–11.6	5.50– 6.00	four-zone reverse geometry	Autorefractor KR8800 cycloplegic autorefraction Lenstar LS 900	10.33 ± 1.65
Zhao, 2021 [38]	Randomized controlled clinical trial, 12 months	Chinese, 20	x	x	x	x	x	x	Autorefractor TOPCON (KR-800) cycloplegic autorefraction Lenstar LS 900	11 ± 1.17

* Dk (oxygen permeability, (10–11 cm^2^ × mL O_2_)/(s × mL × mmHg) or water content).

**Table 3:** Summary of studies included in the meta-analysis comparing orthokeratology and multifocal soft contact lenses.

Author, Year	Study Type, Time	Etnia, Simple Size	Brand	Material	Dk *	Overall Diameter (mm)	Optic Zone (mm)	Geometry	Outcome Assessment Method	Age Mean ± SD (Years)
Huang, 2022 [55],	Prospective, nonrandomized, controlled study	Chinese, 30	Bausch + Lomb	Oprifocon A	127	10.6	6.2	a four-zone reverse-geometry	Cycloplegic Lenstar LS 900	9.90 ± 1.27
Fang, 2022 [31],	Parallel, longitudinal, single-blind, randomized clinical trial, 12 months	Chinese, 20	Paragon CRT, Paragon, Gilbert, AZ)	x	x	9.5–12	5.0/6.0	x	Humphrey Autorefractor Keratom HARK 599 cycloplegic autorefraction IOL Master 700	12.50 ± 0.30
Jiang, 2021 [56]	Nonrandomized controlled clinical trial, 12 months	Chinese, 35	Euclid Systems Corp., Herndon, USA	Oprifocon A	90	10.2–10.6	6.2	a four-zone reverse-geometry	Cycloplegic IOL Master	10.50 ± 1.60

* Dk (oxygen permeability, (10–11 cm^2^ × mL O_2_)/(s × mL × mmHg) or water content).

**Table 4:** Summary of studies included in the meta-analysis comparing orthokeratology and gradient soft contact lenses.

Author, Year	Study Type, Time	Etnia, Simple Size	Brand	Material	Dk *	Overall Diameter (mm)	Optic Zone (mm)	Geometry	Outcome Assessment Method	Age Mean ± SD (Years)
Pauné, 2015 [36]	Prospective, longitudinal, nonrandomized study	Spanish, 18	Precilens, Paris, France	hexafoco n B	141	10.00–11.60	x	x	Grand Seiko Autorefract/Keratom WAM-5500 cycloplegic autorefraction A-scan ultrasonography	12.27 ± 1.76

* Dk (oxygen permeability, (10–11 cm^2^ × mL O_2_)/(s × mL × mmHg) or water content).

**Table 5:** Summary of studies included in the meta-analysis comparing orthokeratology and modified orthokeratology with enhanced peripheral defocus.

Author, Year	Study Type, Time	Etnia, Simple Size	Brand	Material	Dk *	Overall Diameter (mm)	Optic Zone (mm)	Geometry	Outcome Assessment Method	Age Mean ± SD (Years)
Loertscher, 2021 [57],	Randomized, longitudinal controlled clinical trial, 12 months	Chinese, 30	Bausch + Lomb	hexafocon A	100	13	6.0 mm	x	Autorefractor Shin-Nippon K 5001 noncycloplegic autorefraction Lenstar LS 900	12.20 ± 1.30

* Dk (oxygen permeability, (10–11 cm^2^ × mL O_2_)/(s × mL × mmHg) or water content).

**Table 6:** Summary of studies included in the meta-analysis comparing orthokeratology and single-vision spectacles combined with low-dose atropine.

Author, Year	Study Type, Time	Etnia, Simple Size	Brand	Material	Dk *	Overall Diameter (mm)	Optic Zone (mm)	Geometry	Outcome Assessment Method	Age Mean ± SD (Years)
Zhao, 2021 [38]	Randomized controlled clinical trial, 12 months	Chinese, 20	x	x	x	x	x	x	Autorefractor TOPCON (KR-800) cycloplegic autorefraction Lenstar LS 900	11.00 ± 1.17
Du, 2022 [58]	Nonrandomized controlled clinical trial, 12- months	Chinese, 50	ALPHA Corp., Nagoya, Japan	Silicone and Fluorine methacrylate compounds	104	10.5	6.0 mm	reverse geometry	Autorefractor TOPCON (KR-800) cycloplegic autorefraction IOL Master	11.92 ± 2.47

* Dk (oxygen permeability, (10–11 cm^2^ × mL O_2_)/(s × mL × mmHg) or water content).

### 3.8. Overall Comparision

**Figure 4:**
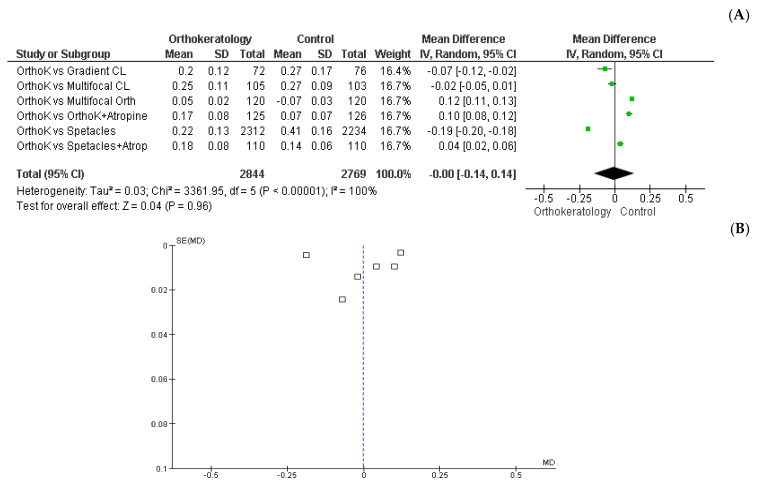
Overall comparison of orthokeratology versus all other myopia control interventions. The forest plot (**A**) shows no significant overall difference in efficacy (mean difference = 0.00 mm; 95% CI: −0.14 to 0.14) across 31 studies, with substantial heterogeneity (I^2^ = 100%). The funnel plot (**B**) demonstrates a relatively symmetric distribution of studies, suggesting limited publication bias despite the marked heterogeneity of interventions and study designs. Green squares represent the effect estimates of the individual studies (with square size proportional to study weight), and black diamonds represent the pooled effect estimates with their 95% confidence intervals. The blue dashed vertical line represents the pooled mean difference (overall effect estimate) for each meta-analysis.

An overall meta-analysis was conducted combining all comparison groups to assess the general position of orthokeratology relative to all other myopia control interventions (Figure 4A). This analysis included all 31 studies and all intervention types: spectacles, orthokeratology plus atropine, multifocal contact lenses, gradient soft contact lenses, enhanced OK, and atropine therapy with single-vision spectacle correction. The pooled mean difference was 0.00 mm (95% CI: −0.14 to 0.14 mm), indicating that when all comparisons are combined, orthokeratology shows equivalent efficacy to the average of all other interventions. However, this overall estimate masks important differences between specific comparisons, as demonstrated in the individual analyses above. Heterogeneity was maximal (I^2^ = 100%, *p* < 0.00001), reflecting the diverse nature of the comparison groups and the varying efficacy of different interventions. This extreme heterogeneity indicates that the overall pooled estimate should be interpreted with caution and that the specific pairwise comparisons provide more clinically relevant information. The key insight from this overall analysis is that orthokeratology occupies a middle position in the spectrum of myopia control efficacy: more effective than single-vision spectacles, equivalent to multifocal contact lenses, but less effective than combination therapies (orthokeratology plus atropine, spectacles plus atropine) and potentially less effective than enhanced optical designs (multifocal orthokeratology). The funnel plot for the overall comparison (Figure 4B) showed wide scatter reflecting the heterogeneity of interventions and study designs, with relatively symmetric distribution around the null effect.

### 3.9. Publication Bias Assessment

Funnel plots were generated for all comparisons to assess potential publication bias (Figure 3). Visual inspection of funnel plots provides a qualitative assessment of whether small studies with null or negative findings may be missing from the published literature. For the orthokeratology versus spectacles comparison (Figure 3A), which included the largest number of studies (*n* = 25), the funnel plot showed relatively symmetric distribution around the pooled effect estimate. While some scatter was observed, particularly among smaller studies, the overall pattern did not suggest major publication bias. The presence of studies with varying effect sizes on both sides of the pooled estimate suggests that studies with different findings have been published. For comparisons with fewer studies (orthokeratology vs. atropine combination, multifocal contact lenses, spectacles plus atropine), funnel plots showed limited scatter due to the small number of included studies. While no obvious asymmetry was detected, the small sample sizes limit the power to detect publication bias through visual inspection. For comparisons based on single studies with multiple time points (gradient soft contact lenses, enhanced OK), funnel plots showed minimal scatter as expected. However, funnel plot analysis is not applicable to these comparisons, as the method requires independent studies to assess publication bias. No conclusions regarding publication bias can be drawn for these two comparisons. The overall funnel plot (Figure 4B) showed wide scatter reflecting the heterogeneity of interventions, with relatively symmetric distribution around the null effect. This symmetry suggests that studies showing both superiority and inferiority of orthokeratology compared to various interventions have been published, reducing concerns about systematic publication bias. Formal statistical tests for funnel plot asymmetry (Egger’s test) were not performed due to the high heterogeneity across studies and the limited number of studies in most comparisons, as these tests have limited validity under these conditions. However, the visual inspection of funnel plots, combined with the inclusion of studies showing varying effect sizes and directions, provides reasonable confidence that major publication bias is not substantially affecting the conclusions of this meta-analysis.

## 4. Discussion

This systematic review and meta-analysis provides comprehensive evidence regarding the comparative efficacy of orthokeratology for myopia control in children and adolescents.

The findings demonstrate that orthokeratology is significantly more effective than single-vision spectacles in slowing axial elongation, with a mean reduction of 0.16 mm over the study periods analyzed. This effect size is clinically meaningful, as modeling studies suggest that even modest reductions in axial elongation during childhood can substantially reduce the lifetime risk of high myopia and associated complications [59]. The subgroup analysis by follow-up duration revealed important temporal patterns, with treatment effects increasing from 0.04 mm at less than 3 months to 0.26 mm at 24 months. This cumulative benefit reflects the sustained slowing of axial elongation with continued orthokeratology wear, rather than a one-time reduction. The annualized effect size of approximately 0.13 mm per year represents a reduction in axial elongation rate of approximately 40–50% compared to spectacles, consistent with previous meta-analyses [17,60]. The significant superiority of orthokeratology over single-vision spectacles supports its use as a first-line optical intervention for myopia control in appropriate candidates. Current evidence indicates that orthokeratology is among the most effective optical treatments available for slowing axial elongation and myopia progression in children, particularly in motivated patients with suitable corneal characteristics [61].

The comparison of orthokeratology to multifocal contact lenses revealed equivalent efficacy, with a mean difference of 0.00 mm and confidence intervals excluding clinically meaningful differences. This finding is consistent with the hypothesis that both interventions work through similar mechanisms involving peripheral myopic defocus [24]. The equivalent efficacy suggests that the choice between orthokeratology and multifocal contact lenses can be guided by patient preferences, lifestyle considerations, and individual suitability rather than expected efficacy differences [62,63]. The comparable efficacy observed between orthokeratology and multifocal soft contact lenses suggests that both modalities represent effective optical interventions for myopia control. Therefore, treatment selection may be guided by patient lifestyle, ocular characteristics, and personal preferences rather than efficacy considerations alone. Multifocal soft contact lenses may be preferred in children who favour daytime lens wear or are unsuitable candidates for orthokeratology, whereas orthokeratology may offer advantages for children wishing to remain spectacle- and contact lens-free during daytime activities, including sports and aquatic activities [31,64].

The finding that orthokeratology combined with low-dose atropine (0.01%) provides superior myopia control compared to orthokeratology alone is particularly important for clinical practice. The additional benefit of approximately 0.09 mm over study periods averaging 12–18 months translates to an additional reduction of approximately 0.06 mm per year, representing a clinically meaningful enhancement. This additive effect supports the hypothesis that optical and pharmacological mechanisms of myopia control are at least partially independent, with atropine likely acting through non-accommodative pathways distinct from the peripheral defocus mechanism of orthokeratology [23,53]. The superior efficacy of orthokeratology combined with low-dose atropine compared with orthokeratology alone supports consideration of combination therapy, particularly for children with rapid myopia progression or those at high risk of developing high myopia. Evidence from recent meta-analyses indicates that the addition of 0.01% atropine provides an additive effect in reducing axial elongation while maintaining a generally favorable safety profile, with only modest increases in pupil diameter and limited effects on accommodation. Therefore, combination therapy may be an appropriate strategy for patients requiring enhanced myopia control beyond that achieved with orthokeratology alone [65,66].

The comparison of orthokeratology to atropine therapy with single-vision spectacle correction revealed that atropine therapy with single-vision spectacle correction was more effective than orthokeratology alone, though the difference (0.04 mm) was smaller than the benefit of adding atropine to orthokeratology (0.09 mm). This pattern suggests that orthokeratology provides some myopia control benefit beyond that of spectacles, but that atropine provides consistent additive benefit regardless of the optical correction method. A hierarchy of efficacy was observed, with conventional spectacle correction showing the least effect on myopia control, followed by orthokeratology and low-dose atropine monotherapies, while the combination of orthokeratology and low-dose atropine demonstrated the greatest reduction in axial elongation. These findings are consistent with previous network meta-analyses reporting a synergistic effect of combined therapy compared with either intervention alone [67].

The observed trend toward greater efficacy with enhanced optical designs may be explained by their ability to modify peripheral retinal image quality and increase myopic defocus across a larger retinal area [68]. Previous studies have demonstrated associations between peripheral refractive patterns, retinal contour, and myopia progression, providing a biological rationale for continued refinement of optical designs aimed at optimizing peripheral defocus profiles [69]. The limited evidence comparing orthokeratology with gradient soft contact lenses and enhanced OK suggests that enhanced optical designs may provide additional benefits beyond conventional orthokeratology. Notably, gradient soft contact lenses and multifocal soft contact lenses share a common design principle of creating peripheral myopic defocus. These two interventions should be interpreted as mechanistically related when considering the overall evidence for soft contact lens-based myopia control strategies. From a clinical perspective, the shared optical mechanism has important implications for treatment selection: in the absence of direct head-to-head comparisons between gradient and multifocal soft contact lenses, clinicians may consider the available evidence for both modalities collectively when counselling patients on daytime soft contact lens-based myopia control. The choice between gradient and multifocal soft contact lens designs should therefore be guided by practical factors such as lens availability, fitting characteristics, cost, and individual patient preference rather than by expected differences in efficacy. However, these findings are currently derived from isolated studies and should be interpreted cautiously until replicated in larger randomized controlled trials. If confirmed, such results would support ongoing innovation in lens design and further optimization of peripheral defocus profiles as a strategy to maximize myopia-control efficacy [10,70].

The availability of multiple evidence-based myopia control interventions allows treatment to be individualized according to patient-specific factors. Clinical guidelines recommend considering age, baseline refractive error, axial elongation rate, risk of high myopia, lifestyle requirements, treatment adherence, and tolerance of potential adverse effects when selecting the most appropriate intervention [64,71]. The increasing treatment effect observed with longer follow-up highlights the importance of maintaining therapy throughout the period of active ocular growth and myopia progression. Evidence from atropine studies has demonstrated rebound progression following treatment cessation, particularly with higher concentrations, emphasizing the need for long-term treatment planning and careful discontinuation strategies [22,72]. Regular monitoring of axial length and refractive error is essential for evaluating treatment efficacy and guiding clinical decision-making. Current guidelines recommend periodic assessment to identify poor responders who may require treatment intensification or combination therapy, while children demonstrating adequate control may continue with their existing regimen. Axial length monitoring is particularly valuable as it provides a sensitive and objective measure of treatment response [71,73].

Several limitations of this meta-analysis should be acknowledged. First, substantial statistical heterogeneity was observed across most comparisons (I^2^ = 83–98%), indicating considerable variability in treatment effects between studies. This heterogeneity likely reflects differences in participant characteristics, including age, baseline refractive error, ethnicity, and risk of progression, as well as variations in intervention protocols, follow-up duration, and study design. Although random-effects models were used to account for between-study variability, some pooled estimates should be interpreted cautiously. Second, several comparisons were based on a limited number of studies, restricting the precision of effect estimates and preventing robust assessment of publication bias or meaningful subgroup analyses. In particular, comparisons involving gradient soft contact lenses and enhanced OK were each represented by a single study and therefore require confirmation in future investigations. Third, follow-up duration varied substantially across studies, ranging from 1 to 24 months. Although time-specific analyses were conducted where possible, differences in treatment duration may have contributed to heterogeneity and complicated direct comparisons between interventions. Furthermore, most studies provided only short- to medium-term follow-up, limiting conclusions regarding long-term efficacy, treatment sustainability, and potential rebound effects following treatment discontinuation. Fourth, most included studies were conducted in East Asian populations, particularly in China, where myopia prevalence and progression rates are among the highest worldwide. Consequently, the generalizability of these findings to other ethnic and geographic populations remains uncertain, as genetic, environmental, and behavioral factors may influence treatment response. Fifth, considerable variation existed within intervention categories. Differences in orthokeratology lens geometry, material properties, and fitting strategies, as well as variations in multifocal contact lens designs and atropine concentrations, may have influenced treatment outcomes and contributed to the observed heterogeneity. This variability also limits the identification of optimal treatment parameters. Sixth, although only prospective longitudinal studies were included, not all studies were randomized controlled trials, introducing potential risks of selection bias and residual confounding. In addition, a formal risk-of-bias assessment using standardized instruments was not performed, limiting the ability to evaluate the influence of study quality on pooled estimates. Finally, this meta-analysis focused primarily on axial length as the outcome measure. While axial elongation is widely regarded as the most objective and clinically meaningful indicator of myopia progression, other important outcomes, including refractive error progression, visual performance, quality of life, treatment adherence, and adverse events, were inconsistently reported and could not be quantitatively synthesized. As a result, the present findings provide a comprehensive assessment of efficacy but a more limited evaluation of safety and patient-centered outcomes. Additionally, the literature search was restricted to PubMed and Google Scholar. Although Google Scholar provides broad coverage across multiple academic databases, the absence of a dedicated search in Scopus, Web of Science, or EMBASE may have resulted in failure to identify some eligible studies. Future reviews should incorporate a broader set of databases to maximise sensitivity. A further limitation is the absence of comparative data between orthokeratology and myopia-control spectacle lenses, such as Defocus Incorporated Multiple Segments (DIMS) and Highly Aspherical Lenslet Target (HALT) designs. These represent a rapidly expanding and clinically accessible treatment category. Although search terms for these interventions were included in the updated search strategy, no eligible head-to-head comparative trials with orthokeratology measuring axial length as an outcome were identified within the search period. Future meta-analyses should prioritise inclusion of such comparisons as the evidence base grows.

This systematic review and meta-analysis provides comprehensive evidence that orthokeratology is an effective intervention for slowing axial elongation in myopic children and adolescents. Orthokeratology significantly reduces axial elongation compared to single-vision spectacles, with a mean reduction of 0.16 mm over study periods and approximately 0.13 mm per year, representing a 40–50% reduction in progression rate. The efficacy of orthokeratology is equivalent to multifocal soft contact lenses, supporting both interventions as effective optical strategies for myopia control. Combination therapy with orthokeratology plus low-dose atropine (0.01%) provides superior myopia control compared to orthokeratology alone, with an additional reduction in axial elongation of approximately 0.09 mm over 12–18 months. This additive benefit supports the use of combination therapy for children with rapid progression or high risk of developing high myopia. Similarly, atropine therapy with single-vision spectacle correction demonstrates superior efficacy to orthokeratology alone, indicating that atropine provides consistent benefit regardless of the optical correction method. The availability of multiple evidence-based interventions allows myopia management to be tailored according to individual patient characteristics and risk profiles. Clinical decision-making should consider factors such as age, baseline refractive error, axial elongation rate, lifestyle requirements, treatment adherence, and risk of developing high myopia. Regular monitoring of axial length and refractive status remains essential for evaluating treatment efficacy and guiding therapeutic adjustments, including escalation to combination therapy in poor responders. The substantial heterogeneity observed across studies highlights the multifactorial nature of myopia progression and supports an individualized approach to treatment selection. Future research should focus on long-term efficacy and safety, optimization of combination therapy protocols, and identification of predictors of treatment response to facilitate more personalized myopia control strategies.

## 5. Conclusions

Orthokeratology is an effective evidence-based intervention for slowing axial elongation in myopic children and adolescents. Its efficacy is superior to single-vision spectacles and comparable to multifocal soft contact lenses, while combination therapy with low-dose atropine provides additional benefit. These findings support the use of orthokeratology, alone or in combination with atropine, as an important strategy for myopia control. Further long-term studies are needed to optimize treatment protocols, evaluate sustained efficacy and safety, and identify predictors of treatment response.

## Data Availability

Data supporting the reported results can be requested from the authors.

## Supplementary Materials

The following supporting information can be downloaded at: https://www.mdpi.com/article/10.3390/jcm15155776/s1, Table S1: PRISMA 2020 Checklist.
